# The Relationship Between Physical Literacy and Physical Fitness in Preschool-Aged Children

**DOI:** 10.3390/healthcare14060708

**Published:** 2026-03-10

**Authors:** Mirela Sunda, Iva Blazevic, Barbara Gilic Skugor

**Affiliations:** 1 Faculty of Kinesiology Osijek, Josip Juraj Strossmayer University of Osijek, 31000 Osijek, Croatia; msunda@kifos.hr; 2 Faculty of Educational Sciences, Juraj Dobrila University of Pula, 52100 Pula, Croatia; 3 Faculty of Kinesiology, University of Split, 21000 Split, Croatia; barbara.gilic@kifst.eu

**Keywords:** physical activity, movement behavior, early childhood, health, motor skills

## Abstract

**Highlights:**

**What are the main findings?**

- Physical fitness improved significantly with age in preschool children, while physical literacy levels remained relatively stable across ages and genders.
- Modest, but meaningful associations between physical literacy and physical fitness were observed, with the strongest relationships evident in 5-year-old children.

**What are the implications of the main findings?**

- The findings provide country-specific evidence to support the integration of physical literacy frameworks into Croatian early childhood education.
- Targeted, age-appropriate movement experiences may help align physical development with motivation, confidence, and understanding of physical activity from an early age.

**Abstract:**

Background/Objectives: Physical literacy (PL) is a multidimensional construct that supports lifelong engagement in physical activity, while physical fitness (PF) represents a key health-related outcome and an important component of PL in early childhood. Evidence on the relationship between PL and PF in preschool-aged children is still limited, particularly in Croatia. Therefore, this study aimed to examine the association between PL and PF in preschool children and to explore age- and gender-related differences. Methods: A total of 103 preschool children (58 girls and 45 boys aged 4–6) participated in this cross-sectional study. PL was assessed using Physical Literacy in Children Questionnaire (PL-C Quest), covering physical, psychological, social, and cognitive domains, while PF and anthropometric variables were measured using the PREFIT test battery. Age and gender effects were analyzed using aligned rank transform ANOVA, while associations between PL domains and PF variables were examined using Spearman’s rank correlations. Results: No significant gender differences were observed in PL or PF variables, except for cardiorespiratory endurance among 6-year-olds. Age had a significant effect on most PF indicators, with moderate-to-large effect sizes, but not on PL scores. Significant associations between PL and PF were age-dependent, with the strongest and most consistent correlations observed in 5-year-old children, particularly between total PL and shuttle run performance (ρ = 0.46, *p* < 0.01). Conclusions: PF improves markedly with age during the preschool period, whereas PL appears relatively stable. Modest, but meaningful associations between PL and PF highlight the importance of early, holistic movement experiences that support not only physical development but also motivation, confidence, and understanding of physical activity. Future studies should investigate the parental influence on children’s PL and PF.

## 1. Introduction

Physical literacy (PL) in early childhood (3–6-year-olds) represents a holistic approach to child development that integrates affective, physical, cognitive, and behavioral components: motivation and confidence, physical fitness (PF), knowledge and understanding, and everyday participation in physical activity [1]. In early childhood, PL is especially important because this is the period in which fundamental movement patterns, the perception of one’s own competence, and interest in active play are formed, which can shape a child’s readiness to engage in various forms of movement and physical activity throughout childhood [2]. Children’s physical activity represents one of the most visible and important indicators of their PL, because through everyday movement, children express their motivation, knowledge, skills, and habits related to an active lifestyle [3,4].

Since regular movement stimulates the development and improvement of the cardiovascular and musculoskeletal systems, physical activity in preschool age directly contributes to the development of PF, which is increasingly viewed as a measurable indicator of a child’s current and future health [5,6]. Research shows that better PF in preschool children is associated with more favorable health outcomes; that is, higher PF scores in preschool age, including cardiovascular endurance, relative strength, and agility, predict lower values of fat mass index and more favorable body composition [7]. In addition, higher overall PF in preschoolers has a strong negative association with body fat and a positive association with muscle mass, which further highlights the importance of PF as a health indicator and the target of early prevention of excess body weight [8].

Considering the theoretical and empirical knowledge about the importance of PL and PF in preschool age, and the fact that PF is considered one of the important components and outcomes of PL and an active lifestyle, there is a need for research that simultaneously includes these constructs in early life. Precisely, despite increasing interest in PL during early childhood, several important gaps remain. First, empirical evidence examining the relationship between multidimensional PL and objectively measured PF in preschool-aged children (4–6 years) is still limited, as most studies have focused either on motor competence or isolated components of PL rather than the full construct [9,10]. Second, the majority of available research originates from Western Europe, North America, Canada or Asian populations, while evidence from Central and Eastern European countries, including Croatia, remains scarce [11,12]. Finally, few investigations have simultaneously analyzed all domains of PL (physical, psychological, social, and cognitive) in relation to health-related PF components during the preschool period [13,14]. Therefore, further investigation is needed to clarify whether PF develops in parallel with motivational and cognitive aspects of PL during early childhood.

Examining the relationship between PL and PF in preschool children provides evidence relevant to health-oriented educational practices aimed at increasing physical activity levels and PF, supporting motor development, preventing obesity, and promoting overall physical and mental well-being. By contributing empirical data from the preschool period, this study aligns with current efforts to evaluate and strengthen successful educational practices for health within physical education and psychomotricity, thereby informing teachers, practitioners, and policymakers involved in early childhood education. Therefore, the aim of this study was to determine the relationship between the level of PL and individual components of PF in preschool-aged children, and to examine age and gender differences. Based on existing theoretical assumptions and findings from international research, we hypothesized that higher levels of PL would be positively associated with more favorable PF indicators, while significant gender differences would not be observed in this age group.

## 2. Materials and Methods

### 2.1. Participants

The study included 123 preschool children (58 girls and 65 boys) aged 4 to 6 years, enrolled in regular early and preschool education programs in the Republic of Croatia. Children were categorized into three age groups: 4-year-olds (*n* = 53), 5-year-olds (*n* = 32), and 6-year-olds (*n* = 38). The mean age of the total sample was 4.90 ± 0.84 years (range: 4–6 years). The mean body height of the participants was 112.69 ± 10.01 cm, and the mean body mass was 20.31 ± 3.56 kg. Children were eligible to participate if they were between 4 and 6 years of age, enrolled in a regular kindergarten program, and had written informed consent provided by a parent or legal guardian. Additionally, participants were required to be in good general health and able to understand and follow simple verbal instructions necessary for completing the testing procedures. Children were excluded from participation if they had diagnosed neurological, musculoskeletal, or developmental disorders that could significantly affect motor performance or physical activity participation. Furthermore, children with chronic diseases limiting physical exertion, acute illness or injury at the time of measurement, or incomplete participation in the assessment protocol were not included in the final analysis. Parents and guardians were informed in advance about the aim and protocol of the study and provided written informed consent prior to data collection. The implementation of the study was approved by the Ethics Committee of the Faculty of Kinesiology in Osijek (Reference No. 2158-110-01-24-85).

### 2.2. Variables and Measurements

The study analyzed morphological, motor, and functional variables, as well as physical literacy variables. Morphological variables included body height, body mass, body mass index, and waist circumference. Motor variables included handgrip strength, explosive strength of the lower extremities, speed/agility, coordination, and balance. The functional variable was cardiorespiratory endurance assessed by the 20 m progressive shuttle run. Physical literacy was assessed using the Australian version of the Physical Literacy in Children Questionnaire (PL-C Quest).

Assessment of morphological characteristics and motor and functional abilities was conducted using the PREFIT test battery for preschool children [15,16].

Body height was measured using a stadiometer. The child stood barefoot, upright, with fully extended legs, arms alongside the body, and gaze directed straight ahead. The result was read from the scale with an accuracy of 0.1 cm. Body mass was measured using a diagnostic scale. The child stood in the middle of the scale without footwear, feet placed on the marked areas, with body weight evenly distributed. The value was read with an accuracy of 0.1 kg. Body mass index (BMI) was calculated as the ratio of body mass (kg) to the square of body height (m^2^). The obtained value was expressed in kg/m^2^ and used as an indicator of the relationship between body mass and height. Because BMI interpretation in children must be age- and sex-specific, weight status was classified using the International Obesity Task Force (IOTF) cut-offs. Overweight and obesity were defined using age- and sex-specific BMI thresholds corresponding to adult BMI values of 25 kg/m^2^ and 30 kg/m^2^ at age 18, respectively [17]. Waist circumference was measured using a measuring tape positioned at the level of the navel. The child stood upright, with the abdomen relaxed to ensure the most accurate measurement possible. The result was read with an accuracy of 0.1 cm.

Handgrip strength was measured using a hand dynamometer. The child stood upright and squeezed the dynamometer maximally with one hand for at least three seconds, ensuring that the device did not touch the body. Two trials per hand were performed, and the better result was used for analysis.

The standing long jump was performed with a two-foot take-off from the take-off line, using an arm swing. After landing, the child maintained the position so that the distance from the take-off line to the nearest point of contact could be measured. The test was repeated three times, and the best result was used in the analysis.

The 4 × 10 m running test was conducted between two parallel lines 10 m apart. The child ran four lengths at maximum speed, crossing the line with both feet at each turn. Time was measured with a stopwatch. Two trials were performed and the better result was used for analysis.

Balance was assessed using the one-leg stance test with eyes open and arms extended. The test ended when the child moved the supporting leg or touched the ground with the raised leg. One trial was performed on each leg, and the average time was used.

In the 20 m progressive shuttle run test, children ran between two lines set 20 m apart in time with an audio signal. The running speed increased at regular time intervals, and the test ended when the child failed twice consecutively to reach the line in time with the signal or stopped due to fatigue. The result was expressed as the last completed level.

The PL-C Quest is a pictorial self-assessment questionnaire designed to assess children’s physical literacy. It includes 30 color pictures depicting characters performing actions and activities and covers four domains of physical literacy (physical, psychological, social, and cognitive). The physical domain assesses children’s perceived physical competence and movement capability, including fundamental movement skills (e.g., running, jumping, throwing, catching), balance and coordination, as well as strength and speed during play and structured movement situations. The psychological domain covers motivation, self-confidence, enjoyment of physical activity, and related aspects. The social domain refers to cooperation, ethics, and social values, while the cognitive domain includes tactical thinking, safety measures, and rules of games. This version of the questionnaire for younger children is administered through an individual conversation between the child and the researcher, who reads the text accompanying the pictures as if telling a story. The child chooses the answer that is “more like me” and then evaluates whether it is “a lot like me” or “a little like me.” The administration time is between 15 and 20 minutes per child, depending on the child’s cooperation. The scoring method is shown in the attached figure. Each domain and the total score are scored separately: physical domain 12–48 points, psychological domain 7–28 points, social domain 4–16 points, cognitive domain 7–28 points, and total score 30–120 points [18].

### 2.3. Research Procedure

The study was conducted during April 2025 in two kindergartens during children’s regular attendance. Measurements were carried out under standardized conditions according to a predefined protocol by trained examiners. The same research activities were conducted in each kindergarten over ten working days and by the same researchers to minimize measurement errors.

Procedures included (in the following order):

- Assessment of physical literacy using the questionnaire,
- Anthropometric testing of children,
- Assessment of physical fitness in children.

Measurements were conducted in the early morning and late morning hours, in classrooms, halls, and outdoor areas of the kindergarten. The last day of each week was reserved for make-up measurements for children who were absent during testing days. All data were collected anonymously and used exclusively for scientific purposes.

### 2.4. Statistical Analysis

Descriptive statistics (mean and standard deviation) were calculated separately for each age group (4-, 5-, and 6-year-olds) and further divided by gender (boys and girls). Prior to inferential analyses, the normality of distribution for all variables was assessed using the Shapiro–Wilk test and confirmed visually through Q–Q plots and histograms. Several variables showed deviations from normality, particularly within smaller age- and gender-stratified subsamples. Therefore, nonparametric methods were applied.

Gender differences within each age group were examined using the Mann–Whitney U test. To assess the effects of age and gender on all outcome variables, a nonparametric two-way aligned rank transform ANOVA (ART ANOVA) was conducted. This method allows for factorial analysis of variance while maintaining the robustness of nonparametric testing. Effect sizes were interpreted using partial eta squared (η^2^), with qualitative magnitudes categorized as small (η^2^ ≥ 0.01), medium (η^2^ ≥ 0.06), or large (η^2^ ≥ 0.14) [19].

Spearman’s rank-order correlation coefficients were used to assess the relationships between physical literacy domains (physical, psychological, social, cognitive, and total score) and selected anthropometric and physical fitness measures. These correlations were calculated separately for each age group, but not across gender due to the absence of significant gender differences in the respective variables. Correlation strength was interpreted according to Cohen’s guidelines: small (ρ ≥ 0.10), medium (ρ ≥ 0.30), and large (ρ ≥ 0.50) [20].

All statistical analyses were performed using Statistica v15 (TIBCO, Palo Alto, CA, USA) with appropriate statistical and visualization libraries. The significance level for all statistical tests was set at *p* < 0.05.

## 3. Results

Descriptive statistics for the total sample, as well as by gender across all age groups (4-, 5-, and 6-year-olds), are presented in Table 1. According to IOTF age- and sex-specific BMI cut-offs, 88.6% of the total sample were classified as normal weight, 8.9% as overweight, and 2.4% as obese. When analyzed by sex, 81.0% of girls were classified as normal weight, 15.5% as overweight, and 3.4% as obese, whereas 95.4% of boys were classified as normal weight, 3.1% as overweight, and 1.5% as obese. There were no statistically significant differences between boys and girls in any of the variables, except in the shuttle run for the 6-year-olds, where boys had better results.

**Figure 1:**
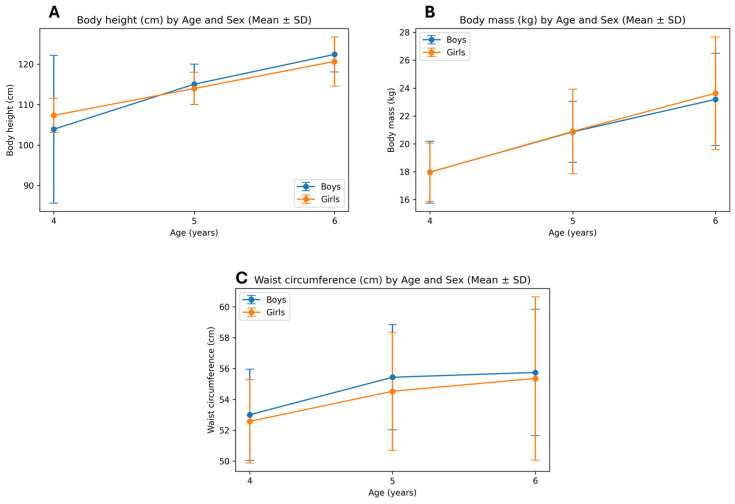
Age-related differences in anthropometric characteristics stratified by sex: (**A**) body height, (**B**) body mass, (**C**) waist circumference. Values are presented as mean ± standard deviation.

**Figure 2:**
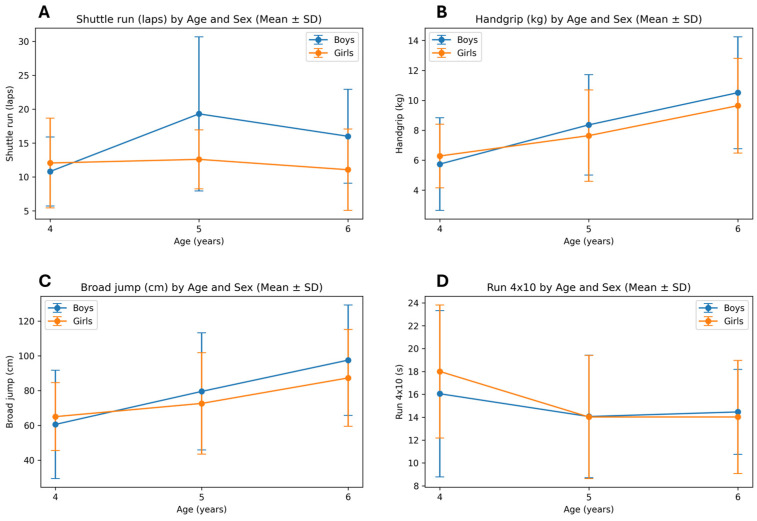
Age-related differences in physical fitness performance stratified by sex: (**A**) shuttle run (laps), (**B**) handgrip strength (kg), (**C**) standing broad jump (cm), and (**D**) 4 × 10 m shuttle run (s). Values are presented as mean ± standard deviation.

Moreover, to better visually present age and gender trends in anthropometric indices, Figure 1 and Figure 2 were created. Trends of PL are not presented graphically as there were no gender nor age differences. In Figure 1, it is visible that body height and body mass increased progressively with age in both boys and girls. No statistically significant sex differences were observed in anthropometric variables across age groups. Waist circumference showed a gradual increase with age in both sexes.

Figure 2 shows significant age-related improvements in shuttle run performance, handgrip strength, and standing broad jump, particularly between ages 4 and 5 years. Boys demonstrated higher values in explosive strength and cardiorespiratory endurance at ages 5 and 6, whereas sex differences were minimal at age 4. Performance in the 4 × 10 m shuttle run improved with age, with lower completion times observed in older children.

The results of the aligned rank transform ANOVA (ART ANOVA) are shown in Table 2. A statistically significant main effect for the factor Age was found for height (large ES), weight (large ES), waist circumference (medium ES), shuttle run (medium ES), handgrip strength (large ES), standing broad jump (large ES), and 4 × 10-m shuttle run (large ES). No statistically significant main effects for the factor Gender were observed. Similarly, interaction effects between Age and Gender did not reach statistical significance for any of the measured variables.

To explore associations between PL and other measured outcomes, Spearman’s rank-order correlations were calculated separately within each age group. Table 3 presents the correlation coefficients (ρ) and corresponding *p*-values for each combination of physical literacy components (PL_Total, PL_Physical, PL_Psychological, PL_Social, PL_Cognitive) and outcome variables (anthropometric and fitness measures). For the 4-year-olds’ group, several statistically significant correlations were observed, including the one between PL_Total and waist circumference (ρ = 0.30, *p* < 0.05), and between PL_Total and handgrip strength (ρ = 0.28, *p* < 0.05). In the 5-year-olds’ group, stronger correlations were observed, with significant associations between PL_Total and shuttle run (ρ = 0.46, *p* < 0.01), and 4 × 10 m run (ρ = 0.44, *p* < 0.01), along with associations between several PL domains and fitness tests. Among 6-year-olds, fewer statistically significant correlations were observed. However, PL_Cognitive showed moderate associations with handgrip strength.

## 4. Discussion

The present study explored the relationship between PL, anthropometric indices and PF among preschool-aged children from Croatia and examined age and gender differences across related domains. The findings revealed that while gender did not significantly influence PL or PF variables, age showed a consistent effect on several fitness indicators, including body height, body mass, handgrip strength, shuttle run, and coordination-based tests. Moreover, the correlational analyses indicated modest yet meaningful associations between components of PL, particularly total PL and its cognitive and social domains, and selected PF measures such as shuttle run performance and handgrip strength, with more notable associations in younger age groups. Therefore, the hypothesis that higher PL would be associated with more favorable PF indicators was partially supported.

### 4.1. Gender Parity in Physical Literacy and Fitness Development

The results of this study showed no statistically significant gender differences in any of the measured variables of PF and PL among preschool-aged children. This finding is consistent with earlier research indicating that at this developmental stage, biological and hormonal differences between boys and girls have not yet resulted in substantial divergence in motor performance or physical capacities [21]. Firstly, no significant gender differences were found in body height, body mass, or waist circumference among preschool-aged children. Importantly, BMI was interpreted using age- and sex-specific IOTF pediatric cut-offs rather than adult thresholds. This can be explained by the general development pattern seen in early infancy, where girls grow slightly faster than boys at first [22]. However, starting around age four, both sexes follow a similar linear growth rate of roughly 5–6 cm per year until puberty [23]. The similar functioning of hormonal regulators during this stage of development, especially the thyroid hormones and the growth hormone/insulin-like growth factor-1 axis, which control somatic and musculoskeletal development, is reflected in this uniformity [21]. Thus, the absence of significant anthropometric differences proves that preschool boys and girls share similar physiological growth conditions, with minimal influence of sex hormones before puberty.

Secondly, there were no gender differences in PF variables among children aged 4 and 5, while there was only a small difference in the shuttle run test between boys and girls aged 6. The general absence of significant gender differences in PF indicates that boys and girls in the preschool period possess comparable levels of motor competence and physiological readiness. This result aligns with previous findings showing that sexual dimorphism in physical performance typically emerges only after the onset of puberty, when hormonal differences, particularly in testosterone and muscle mass development, begin to influence strength and endurance capacities [24,25,26]. Specifically, evidence indicates that the marked male advantage in strength and aerobic performance coincides with pubertal increases in circulating testosterone, which contribute to greater muscle mass, hemoglobin concentration, and oxygen-carrying capacity. In contrast, during early childhood, boys and girls display largely comparable hormonal profiles, and growth and motor development are primarily driven by general maturation processes and environmental influences rather than sex-specific endocrine mechanisms [24,25,26]. At this early stage, neuromuscular coordination and gross motor skills appear to develop primarily through experience, environmental stimulation, parental support and opportunities for active play rather than through biological maturation [21]. Similar results were reported in the PREFIT project, which found minimal gender-related variance in health-related fitness components among children under six years of age [27]. Likewise, it was observed that structured movement interventions improved children’s coordination, balance, and strength equally in both sexes, suggesting that responsiveness to training is not gender-dependent during early childhood [22,28,29]. Taken together, these findings emphasize that differences in PF performance are more likely shaped by levels of engagement, play exposure, and motor learning opportunities rather than innate sex-based physiological distinctions. As researchers who conducted this study are actively working with these children, we observed that these children have the same environment and opportunities for active play, which means that they had similar movement options throughout childhood, which further supports the previous theory for not observing gender differences in PF.

Finally, the lack of gender differences in the physical, psychological, social, and cognitive PL domains implies that boys and girls exhibit similar levels of motivation, self-assurance, and comprehension when it comes to physical activity. Similar PL evaluation instruments, including the PL-C Quest, have also been used in earlier research that found little gender variation in the early years [18,30]. These findings suggest that rather than being the product of intrinsic variations, differences in PL seen in older children may develop later as a result of sociocultural factors and diverse activity preferences [22]. Another potential reason could be the theory that children compare themselves with same-gender peers, which leads to no gender differences in perceived PL. Therefore, through inclusive and equitable physical activity programs, educators and practitioners have an equal opportunity to foster healthy physical habits across genders during the preschool years.

### 4.2. Age-Related Variations in Physical Fitness and Physical Literacy Variables

Numerous PF markers, such as anthropometric characteristics, cardiorespiratory endurance, muscular strength, coordination, and agility, were found to be significantly influenced by age. According to earlier research using the PREFIT battery, this result supports the anticipated developmental trajectory of motor and physiological progress during early life [15,27]. Between the ages of four and six, rapid neuromotor maturation and increasing control over gross motor movements contribute to substantial improvements in balance, jumping, and running performance [21,31]. Increases in body mass, neuromuscular coordination, and muscle strength all promote these gains. Longitudinal studies looking at children’s PF development have shown similar age-related tendencies, emphasizing this phase as a sensitive window for developing motor competence that supports future physical activity participation [32,33]. Although significant age-related increases were observed across most PF indicators, these improvements should be interpreted cautiously. Between ages 4 and 6, substantial interindividual variability in biological maturation exists, and increases in strength, endurance, coordination, and anthropometric measures may largely reflect normative growth and neuromuscular development rather than meaningful differences in health-related fitness per se [21,31]. Therefore, the observed age effects likely represent a combination of maturational processes and accumulated movement experience. The alignment of PF outcomes with PREFIT normative standards further indicates that the observed age-related increases likely reflect typical developmental progression rather than exceptional or clinically concerning deviations in fitness levels.

Interestingly, despite clear improvements in PF, PL scores did not show significant age differences, suggesting that PL development may not parallel the rapid physiological maturation observed in fitness domains. Since PL encompasses affective and cognitive aspects, such as motivation, understanding, and decision-making, it likely develops more gradually and depends on accumulated experiences, guidance, and reflection within structured learning contexts [2]. Previous studies have emphasized that children’s understanding of movement concepts and self-perceived competence often lags behind their physical abilities [30,34]. This indicates that while maturation drives fitness improvements, intentional pedagogical strategies are necessary to foster the cognitive and psychological components of PL. As such, preschool education should emphasize experiential learning and reflective play to align children’s growing physical capacities with their awareness, motivation, and confidence to move effectively and safely.

Moreover, children’s self-perceptions of their movement competence are molded by comparisons with peers of the same age rather than by an objective developmental standard, which is another explanation for the lack of age-related differences in PL [35,36]. Given that the PL-C Quest is a self-assessment tool, children are likely to compare their skills and abilities to those of others in their immediate social milieu who have similar developmental traits, usually their classmates or playmates. Even when actual motor skill or physical fitness increases with age, this propensity for age-referenced self-evaluation may make it more difficult to identify distinctions between age groups. Therefore, the consistent PL ratings shown in this study may be partially explained by children’s perception of their competence as stable throughout time, despite quantifiable age-related advances in physical performance.

### 4.3. Associations Between Physical Literacy and Physical Fitness

The associations observed between PL and PF in this study were generally small to moderate, suggesting that although these constructs are meaningfully connected in early childhood, they are far from identical. In practical terms, being physically fit does not automatically mean that a child feels confident, motivated, or socially engaged in movement contexts. This is consistent with the understanding of PL as a broad and multidimensional concept that extends beyond physical performance to include psychological, cognitive, and social elements [1,14]. Interestingly, the moderate association found between total PL and cardiorespiratory endurance in 5-year-old children may point to a developmental stage in which children’s self-perceptions begin to more closely reflect their actual physical capabilities. Around this age, increasing cognitive awareness and social comparison may allow children to form slightly more accurate judgments about their own competence.

At the same time, caution is warranted when interpreting these relationships. The preschool years are marked by rapid and uneven biological development. Improvements in strength, coordination, and endurance between ages 4 and 6 are strongly influenced by normative growth, neuromuscular maturation, and accumulating movement experiences [26]. Moreover, considerable interindividual variability in growth tempo and maturation exists during this period, meaning that some of the observed PL–PF associations may reflect shared developmental processes rather than direct influence of one construct on the other [21,23]. The lack of strong and consistent correlations across all PL domains further reinforces the idea that PL cannot be reduced to PF alone. Motivation, enjoyment, confidence, and understanding of movement rules are central components of PL, and these may develop somewhat independently of measurable fitness performance. Taken together, the findings suggest that PL and PF in preschool children evolve in parallel, shaped by a complex interaction of biological maturation, daily movement opportunities, and learning experiences, rather than through a simple or linear developmental pathway. Importantly, the observed associations between PL and PF should be interpreted as correlational. Given the rapid developmental changes occurring during the preschool years, these relationships may partly reflect shared underlying maturation processes rather than direct causal influence.

Beyond developmental factors, environmental context may also help explain some of the observed patterns. Indeed, Croatian preschool children are exposed to substantial outdoor play time, but also relatively high screen time levels at the national level [4]. Although these broader behavioral patterns were not directly measured in the present study, the participating children were drawn from similar educational settings and shared comparable daily routines within their kindergartens. Such environmental homogeneity, including structured schedules, similar movement opportunities, and comparable pedagogical approaches, may have reduced variability in both PL and PF outcomes. Moreover, since early childhood PL development is strongly influenced by family habits, access to play environments, and movement exposure, relatively similar contextual conditions could partly explain the absence of gender differences and the limited variability observed across PL domains [37]. In this sense, the findings may reflect a relatively uniform developmental environment rather than purely individual-level differences.

### 4.4. Practical and Educational Implications for Early Childhood Programs

The findings of this study offer valuable guidance for shaping early childhood education and health programs. The lack of gender differences points to an important opportunity: boys and girls in preschool can benefit equally from shared, inclusive activities that nurture both PL and PF. This period of life is ideal for introducing a wide range of movement experiences that build coordination, strength, and balance while keeping children engaged and enjoying physical activity. Activities such as cooperative games, obstacle challenges, or rhythm and dance not only strengthen fundamental movement skills but also support social interaction and cognitive growth. Embedding PL frameworks into preschool curricula encourages a more holistic approach to development, one that helps children gain not just physical ability, but also the motivation, confidence, and understanding needed to value and sustain an active lifestyle.

From a broader perspective, the results highlight how important it is to empower both educators and parents to create environments that inspire movement from an early age. Parent-focused programs can demonstrate how involving families can extend the benefits of active play beyond the classroom and help establish healthy daily habits. Although children’s fitness improves naturally with age, providing regular and well-structured opportunities for movement remains crucial. Continuous assessment using tools like the PREFIT battery can also help educators identify developmental needs and guide early interventions. Ultimately, integrating PL principles into early education policy can play a key role in counteracting sedentary lifestyles and childhood obesity, supporting healthier, more active trajectories into later life.

### 4.5. Limitations and Future Research

Several limitations should be considered when interpreting the findings of this study. First, the cross-sectional design does not allow conclusions about causality. Although associations between PL and PF were identified, it remains unclear whether higher PL contributes to better PF, whether children with higher PF perceive themselves as more physically literate, or whether both develop simultaneously. Longitudinal research is needed to clarify these relationships over time. Second, the sample was relatively small and drawn from a limited geographic area, which may reduce the generalizability of the results. Future studies should include larger and more diverse samples to confirm the present findings. PL was assessed using a self-report pictorial questionnaire. While appropriate for preschool-aged children, self-perceptions at this age may not always accurately reflect actual competence. Combining subjective assessments with objective measures of motor competence would provide a more comprehensive evaluation of PL. In addition, habitual physical activity and environmental factors such as parental influence and preschool context were not examined. Given their potential role in shaping both PL and PF, future studies should adopt broader, longitudinal approaches that include these variables.

Importantly, biological maturation was not directly assessed in the present study. Variability in growth tempo and neuromuscular maturation may have influenced PF outcomes, potentially confounding the interpretation of age-related differences. Future studies should consider incorporating maturity indicators or longitudinal tracking to disentangle maturational effects from experiential development. Moreover, future studies should investigate the parental influences on children’s PL, PF and physical activity. Finally, future research should explore age-specific developmental windows to better understand why associations between physical literacy and fitness appeared stronger in certain age groups, particularly among 5-year-olds in the present study. Investigating psychological and cognitive maturation processes alongside physiological development may provide deeper insight into these patterns.

It should also be acknowledged that the researchers were involved in the educational environment in which data collection took place. Although standardized protocols were strictly followed and assessments were conducted objectively, prior familiarity between researchers and children may have influenced children’s comfort levels or responses during PL self-assessment. While such familiarity may have facilitated cooperation and reduced anxiety, its potential influence on self-perceived responses cannot be entirely excluded.

## 5. Conclusions

This study examined the relationship between PL and PF among preschool-aged Croatian children, providing new insights into early developmental patterns. The results showed that while age was significantly related to improvements in fitness components such as strength, endurance, and coordination, PL levels remained relatively stable across age groups. No gender differences were observed in either PL or PF, suggesting that during the preschool years, boys and girls develop under similar physiological and environmental conditions. These findings emphasize that PL, unlike PF, depends more on accumulated experiences, motivation, and learning opportunities than on biological maturation alone. Modest but meaningful associations between PL and selected PF components suggest that these constructs are related during early childhood, although they remain distinct and influenced by multiple developmental and environmental factors. Given the cross-sectional design and the rapid maturational changes characteristic of this age period, the findings should be interpreted cautiously.

From a practical standpoint, the results highlight the importance of creating early educational environments that integrate play, movement, and reflection to nurture both physical competence and a positive attitude toward physical activity. Early childhood educators and families should work together to promote active play and develop children’s confidence, enjoyment, and understanding of movement as part of everyday life. Future research should continue exploring how structured interventions and longitudinal tracking of PL and PF contribute to children’s long-term health and engagement in physical activity, particularly across diverse cultural and educational contexts.

## Figures and Tables

**Table 1:** Descriptive parameters and gender differences in all study variables according to age groups.

4-Year-Olds (*n* = 53)								
	Total		Boys (*n* = 30)		Girls (*n* = 23)		Mann–Whitney U Test	
Variables	Mean	SD	Mean	SD	Mean	SD	U Value	*p*-Level
Body height (cm)	105.41	14.01	103.93	18.26	107.34	4.20	318.00	0.63
Body mass (kg)	17.97	2.15	17.97	2.22	17.96	2.09	342.00	0.96
Waist circumference (cm)	52.81	2.83	53.00	2.96	52.57	2.70	301.00	0.44
Shuttle run (laps)	11.33	5.76	10.80	5.09	12.05	6.63	308.00	0.69
Handgrip (kg)	5.96	2.73	5.74	3.09	6.28	2.12	379.00	0.85
Broad jump (cm)	62.37	26.94	60.56	31.12	65.04	19.56	365.00	0.68
Run_4 × 10 (s)	16.84	6.73	16.05	7.27	18.00	5.82	381.50	0.88
PL_Total	86.20	31.48	81.71	35.87	92.75	22.84	302.00	0.26
PL_Physical	36.11	9.30	34.75	10.76	38.00	6.56	345.50	0.71
PL_Psychological	22.05	5.23	21.31	6.47	23.09	2.50	353.00	0.80
PL_Social	13.24	3.62	12.81	4.34	13.83	2.23	329.00	0.51
PL_Cognitive	21.07	5.76	20.50	6.61	21.87	4.33	364.50	0.40
5-year-olds (*n* = 32)								
	Total		Boys (*n* = 16)		Girls (*n* = 16)			
	Mean	SD	Mean	SD	Mean	SD		
Body height (cm)	114.51	4.47	115.04	4.98	113.98	3.98	106.00	0.42
Body mass (kg)	20.88	2.60	20.86	2.18	20.89	3.03	119.00	0.75
Waist circumference (cm)	54.98	3.60	55.43	3.41	54.52	3.83	109.50	0.50
Shuttle run (laps)	15.85	9.06	19.31	11.40	12.59	4.36	87.00	0.08
Handgrip (kg)	8.00	3.18	8.36	3.35	7.64	3.05	127.50	0.28
Broad jump (cm)	76.20	31.27	79.56	33.65	72.65	29.13	111.00	0.17
Run_4 × 10 (s)	14.04	5.29	14.06	5.36	14.02	5.38	138.50	0.64
PL_Total	86.36	33.23	85.67	33.30	87.06	34.11	113.50	0.60
PL_Physical	36.97	7.00	36.19	5.98	37.75	8.00	113.00	0.58
PL_Psychological	22.66	3.98	22.69	4.41	22.62	3.65	127.00	0.98
PL_Social	14.25	2.33	14.38	2.00	14.12	2.68	126.00	0.95
PL_Cognitive	23.28	3.52	23.12	4.08	23.44	2.99	153.50	0.80
6-year-olds (*n* = 38)								
	Total		Boys (*n* = 19)		Girls (*n* = 19)			
	Mean	SD	Mean	SD	Mean	SD		
Body height (cm)	121.54	5.30	122.41	4.33	120.66	6.10	154.00	0.45
Body mass (kg)	23.41	3.65	23.19	3.31	23.63	4.05	152.00	0.41
Waist circumference (cm)	55.54	4.68	55.74	4.10	55.35	5.30	158.50	0.53
Shuttle run(laps)	13.67	6.89	16.00	6.94	11.06	6.00	94.50	0.04
Handgrip (kg)	10.08	3.45	10.51	3.74	9.64	3.16	180.00	0.60
Broad jump (cm)	92.42	29.95	97.55	31.77	87.30	27.87	150.00	0.18
Run_4 × 10 (s)	14.24	4.32	14.46	3.71	14.02	4.94	188.50	0.77
PL_Total	92.12	24.99	90.30	23.92	93.95	26.50	136.50	0.20
PL_Physical	37.05	7.05	35.89	6.20	38.21	7.80	145.00	0.31
PL_Psychological	23.05	3.62	22.84	2.75	23.26	4.39	159.00	0.54
PL_Social	14.24	2.01	14.11	1.94	14.37	2.11	161.00	0.58
PL_Cognitive	22.63	3.57	22.21	3.43	23.05	3.75	162.50	0.32

Legend: PL—physical literacy; SD—standard deviation.

**Table 2:** Results of the aligned rank transform (ART) ANOVA for study variables by age and gender.

	Main Effects						Interaction Effects		
	Gender			Age			(Gender × Age)		
Variable	F	*p*	μ^2^	F	*p*	μ^2^	F	*p*	μ^2^
Body height	0.81	0.37	0.002	106.69	0.001 **	0.64	0.71	0.49	0.004
Body mass	0.06	0.82	0.001	55.25	0.001 **	0.49	0.06	0.94	0.001
Waist circumference	1.69	0.20	0.01	6.54	0.002 *	0.10	0.02	0.98	0.001
Shuttle run	3.32	0.07	0.03	5.39	0.01 *	0.08	2.59	0.08	0.04
Handgrip	1.39	0.24	0.01	44.71	0.001 **	0.41	0.22	0.80	0.002
Broad jump	1.60	0.21	0.01	22.46	0.001 **	0.25	0.64	0.53	0.01
Run_4 × 10	0.26	0.61	0.01	16.70	0.001 **	0.21	0.08	0.93	0.001
PL_Total	1.82	0.18	0.01	0.22	0.81	0.003	0.12	0.89	0.002
PL_Physical	3.53	0.06	0.03	0.05	0.96	0.001	0.03	0.97	0.001
PL_Psychological	0.33	0.56	0.003	0.50	0.61	0.01	0.60	0.55	0.01
PL_Social	0.22	0.64	0.002	1.27	0.29	0.02	0.09	0.91	0.002
PL_Cognitive	0.69	0.41	0.01	1.68	0.19	0.03	0.08	0.93	0.001

Legend: PL—physical literacy; μ^2^—Partial eta squared; * *p* < 0.01; ** *p* < 0.001.

**Table 3:** Spearman’s rank-order correlations between physical literacy variables and anthropometric and physical fitness variables (total sample and age-stratified).

	Total Sample				
Variable	PL-Physical	PL-Psychological	PL-Social	PL-Cognitive	PL Total
Body height	−0.01	0.10	0.07	0.15	0.08
Body mass	−0.02	0.13	0.08	0.16	0.09
Waist circumference	−0.04	0.06	0.08	0.04	0.05
Shuttle run	0.16	0.11	0.29 **	0.25 **	0.23 **
Handgrip	0.01	0.07	0.12	0.23 **	0.24 **
Broad jump	0.02	0.08	0.09	0.14	0.23 **
Run_4 × 10	0.01	−0.15	−0.20 *	−0.21 *	0.12
	4 years				
	PL-physical	PL-psychological	PL-social	PL-cognitive	PL total
Body height	0.17	0.13	0.10	0.08	0.17
Body mass	0.10	0.15	−0.05	0.01	0.10
Waist circumference	0.17	0.18	0.13	0.21	0.29 *
Shuttle run	0.20	0.11	0.21	0.23	0.25
Handgrip	0.19	0.05	0.20	0.28 *	0.28 *
Broad jump	0.08	0.00	−0.01	0.11	0.21
Run_4 × 10	−0.16	−0.21	−0.19	−0.25	−0.04
	5 years				
	PL-physical	PL-psychological	PL-social	PL-cognitive	PL total
Body height	−0.05	0.06	−0.27	0.20	0.01
Body mass	−0.05	0.08	−0.15	0.08	−0.01
Waist circumference	−0.18	0.00	0.02	−0.17	−0.18
Shuttle run	0.35 *	0.42 *	0.54 **	0.30	0.46 **
Handgrip	−0.19	−0.05	−0.26	0.12	0.19
Broad jump	−0.22	0.09	0.04	0.00	0.21
Run_4 × 10	0.30	−0.09	−0.27	0.11	0.44 **
	6 years				
	PL-physical	PL-psychological	PL-social	PL-cognitive	PL total
Body height	−0.01	−0.07	0.05	0.07	0.01
Body mass	0.04	−0.05	0.19	0.24	0.12
Waist circumference	−0.12	−0.14	−0.05	−0.17	−0.13
Shuttle run	−0.03	−0.16	0.07	0.15	0.01
Handgrip	0.02	0.08	0.10	0.14	0.18
Broad jump	0.14	−0.04	0.03	0.02	0.16
Run_4 × 10	−0.04	−0.04	−0.09	−0.27	0.10

* Denotes *p* < 0.5; ** denotes *p* < 0.01.

## Data Availability

Data are available upon reasonable request due to ethical restrictions.

## Abbreviations

The following abbreviations are used in this manuscript:

PL	Physical literacy
PF	Physical fitness
PL-C Quest	Physical Literacy in Children Questionnaire
PREFIT	Tool for assessing health-related components of PF in children
